# The Interplay Between Social Factors and Metabolic Bariatric Surgery Outcomes—A 5‐Year Prospective Study

**DOI:** 10.1002/osp4.70068

**Published:** 2025-03-24

**Authors:** Uta Sailer, Fatemeh Asgari, Jon A. Kristinsson, Ingela Lundin Kvalem

**Affiliations:** ^1^ Faculty of Medicine Department of Behavioural Medicine Institute of Basic Medical Sciences University of Oslo Oslo Norway; ^2^ Department of Endocrinology, Obesity and Nutrition Vestfold Hospital Trust Tønsberg Norway; ^3^ Center for Morbid Obesity and Bariatric Surgery Oslo University Hospital Oslo Norway; ^4^ Department of Psychology University of Oslo Oslo Norway

**Keywords:** mental health, metabolic bariatric surgery (MBS), post‐surgery outcomes, social relationships, social support

## Abstract

**Introduction:**

Social relationships and support are vital for well‐being and health. In the context of metabolic bariatric surgery (MBS), these relationships might influence emotion regulation and health behaviors that affect surgical outcomes. Conversely, the outcomes of MBS can impact an individual's social dynamics due to enhanced social engagement or potential shifts in social structures resulting from changes in self‐perception.

**Objective:**

This study investigated the bidirectional relationship between social factors and metabolic bariatric surgery (MBS) outcomes, testing if social factors are associated with improved MBS outcomes, and if MBS outcomes are associated with changes in social relationships.

**Methods:**

Patients reported about structural and functional aspects of their social relations before surgery and at 1‐, 3‐ and 5‐year post‐surgery. Physiological and mental health parameters were also collected. The relationship between pre‐surgery social factors and MBS outcomes 5 years post‐surgery was tested with a path model based on regression analysis. The relationship between MBS outcomes 1‐year post‐surgery and social relationships 3 years post‐surgery was tested with multiple and logistic regressions.

**Results:**

Non‐family support predicted higher satisfaction with surgery. Increased family coherence in partnered patients was linked to a greater reduction in blood pressure 5 years post‐surgery. However, relationship stability, social competence, relationship satisfaction, and weight‐loss specific support were not related to biopsychosocial outcomes.

On the other hand, changes in social relationships post‐surgery were not related to weight loss or other outcomes. Depression and anxiety symptoms 1 year after surgery were associated with decreased relationship satisfaction 3 years post‐surgery.

**Conclusion:**

Overall, social factors had limited and selective predictive value to outcomes, potentially due to the dominant influence of physical changes or generally satisfactory relationships. These findings offer insights for patients and healthcare providers on the nuanced implications of MBS beyond physical health.

## 1 Introduction

Human well‐being relies on social relationships, which fulfill a crucial function in promoting and sustaining good health [1]. On the other hand, social isolation and loneliness are related to an increased risk of mortality (see Ref. [2] for a meta‐analysis). Social isolation refers to an objective lack of social contacts [3, 4], whereas loneliness refers to the subjective and troubling feeling that comes with the perception of having fewer social relationships than desired [5, 6]. The concepts illustrate the difference between structural and functional aspects of social relationships (e.g.; [7, 8, 9]). Structural aspects relate to the existence of relationships, such as having a partner or a social network. Functional aspects relate to how the relationships are perceived, the availability of support, and the feelings of belonging and intimacy.

One of the mechanisms behind the negative effects on health may be that social isolation and loneliness promote unhealthy behaviors such as physical inactivity [10, 11] and maladaptive eating [12]. Perceived social isolation is also related to higher fat mass percentage, diminished diet quality, enhanced maladaptive eating behaviors (e.g., cravings), and altered neural responses to food stimuli [13]. Furthermore, social isolation was associated with reduced medication adherence in a study with Chinese older adults with chronic diseases [14]. Social support and loneliness served as key mediating factors in this association. These interconnected social and physiological mechanisms underscore the potential pathways through which social isolation may contribute to increased health risks and the development of obesity.

Loneliness is indeed associated with a higher risk for obesity [15, 16]. A large cohort study from the United Kingdom found that social isolation and loneliness were significantly associated with all‐cause mortality in individuals with obesity [17]. The authors suggested possible explanations for these associations, including the link between social isolation, loneliness and mental health problems in people with obesity. Additionally, they proposed that a lack of social support may worsen health‐risk behaviors such as smoking, physical inactivity, and unhealthy diets, while also reducing adherence to medical recommendations.

Whereas metabolic bariatric surgery (MBS) is currently the most effective treatment for severe obesity (e.g.; [18, 19, 20, 21]), many patients do not achieve substantial weight loss or experience recurrent weight gain over time [22, 23, 24]. This variability in surgical outcomes underscores the need to identify psychological, social, and physiological factors that can predict and potentially improve various surgery outcomes.

Structural aspects of social relationships might be among these factors. An earlier meta‐analysis concluded that post‐operative support groups improved weight loss, but the findings for marital status as a potential proxy for social support were inconclusive [25]. However, a later study found that the odds for weight loss after surgery were seven times higher for patients who were married [26]. A recent study found no difference in weight loss 4 years post‐surgery between patients who were single and those who had a partner prior to surgery [27].

In addition to structural aspects, functional aspects of social relationships seem to also play a role. For example, greater subjectively reported social support was associated with greater weight loss both before and after surgery in a group of 65 bariatric surgery patients [28]. In patients that maintained a relationship from pre‐surgery to five [29] and eight [30] years later, relationship satisfaction was strongly associated with weight loss maintenance. In the latter study, only marital satisfaction had this effect, not perceived support from the family.

Other studies have investigated the effect of support groups and friends. Frequent users of a Facebook support group lost more weight than infrequent users or the control group up to 2 years postoperatively [31]. Similarly, contact frequency with friends and emotional support prior to surgery were positively related to weight loss up to 2 years postoperatively [32]. On the other hand, an RCT comparing a patient group receiving support from a psychologist with a control group receiving usual care found no differences between groups on weight loss 1 year after surgery [33]. These different results might be due to the different measures used, or to perceived support having a larger effect on weight loss than the objective presence of support.

Investigating both functional and structural social aspects, such as support group attendance, marital status, and the size of the weight‐related emotional support network, only the latter predicted weight change over 1 year [34]. However, this effect was mediated by stress: a large support network was only positively associated with weight maintenance for patients with high stress, while patients with low stress and a large support network regained more weight. The authors interpreted these effects as dependent on individual needs, suggesting that a high level of support that is not needed may lead to worse weight outcomes [34]. A few studies specifically looked at how support for healthy eating was related to weight loss. In one of these studies, higher social support for healthy eating habits from friends and family predicted lower weight loss 3‐months post‐surgery [35].

In addition to studies investigating how social support and social relations are associated with the outcome of bariatric surgery, the effect in the opposite direction is also of interest. Indeed, an increasing number of studies investigates how the outcomes of bariatric surgery are associated with social factors in the post‐surgery period. Bariatric surgery has been reported to lead to stable or improved partner relationships 3 years post‐surgery [36, 37]. Similarly, the incidence of marriage and new relationships was higher among individuals at a median of 10 years post‐bariatric surgery compared to obese individuals who did not undergo the procedure [38]. However, the incidence of divorce or separation was also higher in patients who had undergone bariatric surgery [38]. A similar pattern was observed in a different group of patients who had undergone surgery, compared to healthy controls, over a period of 5 years [38]. The increased rate of separation has been attributed to the fact that relationships that were already strained before surgery can become even more strained due to the changes induced by the surgery [37, 39, 40].

In addition to structural changes in relationships following bariatric surgery, functional changes have also been observed. For example, in a sample of patients with an average of 8 years post‐surgery, most reported stable or improved relationship quality [30]. Among those who maintained a relationship, sustained weight loss was associated with greater relationship satisfaction. The authors suggest that relationship quality may play a crucial role in maintaining health behaviors beneficial to weight management. Regarding social behavior, a long‐term study found that surgical patients reported improvements in social interactions over the 15 years following surgery. These patients participated in more group activities and reduced their self‐isolation from family members, unlike control patients who did not experience these changes [41]. Spending more time in social activities was also reported by a different group of patients 5 years post‐surgery [42]. One qualitative study reported that patients were able to make new friends in the clinic and during the treatment [43]. MBS can also require relationship adjustments [43]. For example, some patients reported that friends and family members with overweight distanced themselves from them [44].

Based on the literature suggesting that social relationships might facilitate weight loss and that weight loss might change social relationships, the current longitudinal study aimed to explore both questions simultaneously. In addition, while most existing studies concentrate on weight loss and weight maintenance as primary outcomes, this study aimed to examine additional variables—including satisfaction with weight loss, blood pressure, depression and anxiety, and relationship satisfaction—to provide a more comprehensive understanding of outcomes on biological, psychological, and social levels. Addressing the relationship between structural and functional social factors and biopsychosocial surgical outcomes after MBS in two directions, the following primary hypotheses were tested: Firstly, that positive social relations prior to surgery predict positive biopsychosocial surgical outcomes (including weight loss) 5 years post‐surgery. Secondly, that positive biopsychosocial surgical outcomes 1‐year post‐surgery predict improved social relations 3 years post‐surgery.

## 2 Methods

### 2.1 Participants and Procedure

The data were collected in the framework of the Oslo Bariatric Surgery Study (OBSS) [45, 46], a prospective study conducted in collaboration between the Department of Psychology, University of Oslo (UiO) and the Center for Morbid Obesity, Oslo University Hospital (OUS). The study included patients undergoing either sleeve gastrectomy or Roux‐en‐Y gastric bypass from 2011 to 2013. The patients were assessed at five time points: prior to surgery, and 1‐year, 3, 5 and 10 years after surgery. In the current study, only data up until 5 years were used.

During the recruitment period, 728 patients underwent surgery, of whom 222 were excluded due to enrollment in other ongoing studies. Of the remaining 506 patients, 332 consented to participate, and 302 completed the pre‐surgery questionnaire. Prior to surgery, 16 participants withdrew their consent or did not undergo the procedure, leaving a sample of 316 consenting participants. One year after surgery, 258 of the 316 participants (82%) returned the questionnaire. At three years post‐surgery, 200 of 307 remaining participants (65%) completed the questionnaire, and at five years post‐surgery, 224 of 302 participants (74%) returned the questionnaire. The patients completed the paper questionnaires at home and submitted them via postal mail.

There was no evidence of attrition bias (see Table 1).

**TABLE 1:** Logistic regression model assessing the relationship between pre‐surgery variables and dropouts.

Predictor	Estimate	Std.Error	*z*‐value	Pr (> |*z*|)
Intercept	0.376	2.989	0.126	0.900
Gender	−0.190	0.687	−0.277	0.782
Depression and anxiety prior to surgery	0.047	0.039	1.216	0.224
Relationship satisfaction with partner prior to surgery	−0.147	0.223	−0.659	0.510
Relationship status [with partner] prior to surgery	−1.814	1.057	−1.716	0.086
Social competence	−0.156	0.349	−0.449	0.654
Social resources	0.471	0.470	1.003	0.316
Systolic blood pressure prior to surgery	−0.016	0.015	−1.046	0.295
Expectations of optimal outcome prior to surgery	0.206	0.229	0.900	0.368
Emotional expectations prior to surgery	−0.134	0.264	−0.509	0.611

To test whether social relations prior to surgery predict biopsychosocial surgical outcomes, the time points prior to surgery and 5 years post‐surgery were used, as the variable satisfaction with surgery was only available 5 years post‐surgery. To test if biopsychosocial surgical outcomes predict social relations later on, the time points 1‐year and 3‐year post‐surgery were used, because relationship satisfaction was not assessed at 5 years post‐surgery. For an overview over the available variables at each time point, see Table 2.

**TABLE 2:** Variables collected at the different time points of the study. The number in brackets indicates if the variable measured at this particular time point was used to test whether social relations prior to surgery predict biopsychosocial surgical outcomes (1), or whether biopsychosocial surgical outcomes predict social relations later on (2).

Prior to surgery	1‐year post‐surgery	3‐year post‐surgery	5‐year post‐surgery
Relationship status (1)	Relationship status	Relationship status	Relationship status (1)
Relationship satisfaction (1, 2)		Relationship satisfaction (2)	
Family coherence (1)			
Social resources (1)			
Social competence (1)			
	Weight‐loss specific social support (1)		
Weight (1, 2)	Weight (2)	Weight	Weight (1)
		Recurrent weight gain	Recurrent weight gain (1)
			Satisfaction with surgery (1)
Blood pressure (1)	Blood pressure	Blood pressure	Blood pressure (1)
Depression and anxiety (1)	Depression and anxiety (2)	Depression and anxiety	Depression and anxiety (1)
Emotional expectations (1)			
Expectations of optimal outcome (1)			
Gender (1, 2)	Gender	Gender	Gender
	Satisfaction with weight loss (2)	Satisfaction with weight loss	
		Change in relationship with friends (2)	
	Negative side effects (2)	Negative side effects	

The Regional Research Ethics Committee (2009/1248a) and the Norwegian data protection authority at the Oslo University Hospital (19‐2010 OUS‐A) approved the study. The participants received written and oral information about the study before signing a consent form.

### 2.2 Measures

#### 2.2.1 Variables for Testing if Social Relations Prior to Surgery Predict Biopsychosocial Surgical Outcomes 5 years Post‐Surgery

Five measures of relationship status and support were used. The first measure was relationship status. Patients reported their relationship status pre‐surgery by choosing between three different options: Not married and no partner, not married with partner, or married. The second measure was relationship stability. Relationship stability was derived from relationship status pre‐surgery and 5 years post‐surgery. Three categories were created: “partnered” (partnered at both timepoints), “single” (single at both timepoints), and “changing” (partnered at one time point but not another). Note that for patients reporting to be married or having a partner at two consecutive time points, it is unknown if it was the same partner or not.

The third measure was relationship satisfaction, which was measured prior to surgery and 3 years post‐surgery using the Relationship Assessment Scale (RAS; [47]). The RAS‐items are answered on a 7‐point scale with answer categories ranging from “little” [1] to “much” [7]. An example item of the RAS is “How well does your partner meet your needs?”. The RAS measures a single factor and demonstrates high reliability (Cronbach's alpha = 0.86). It correlates with relevant relationship measures, such as *r* = 0.80 with the well‐established Dyadic adjustment scale [48], and effectively discriminates between couples who stayed together and those who separated [47]. In the current sample, the six‐item RAS‐scale had a Cronbach's alpha of 0.85 at the pre‐surgery timepoint.

Fourth, family coherence, social competence, and social resources, were measured using the Resilience Scale for Adults [49, 50] prior to surgery. The scale evaluates five factors, with three being particularly relevant to the present research questions. The first factor, family coherence, refers to shared familial values, views, and appreciation within the family unit. The second factor, social resources, pertains to the availability and support provided by individuals outside the family. Lastly, social competence addresses the individual's ability to form friendships and feel comfortable in various social contexts. The items are answered on a 5‐point semantic differential scale, with polar answer options at the extremes (e.g., “Meeting new people is [difficult for me ‐ something I am good at]”). The mean of these answers is calculated per factor, ranging from 1 to 5, where higher scores indicate higher levels of family coherence, social resources and social competence. The reliability of these subscales is *α* = 0.87 for family coherence and *α* = 0.83 for social competence and social resources. The subscales correlate positively with a measure of psychological adjustment (correlations from *r* = 0.44 to 0.75) and negatively with an inventory of psychiatric symptoms (correlations from *r* = −0.32 to −0.61). The subscales family coherence and social competence differentiated between healthy participants and patients with different psychiatric diagnoses, but the subscale “social resources” did not [49]. In the current sample, Cronbach's alpha for family coherence was *α* = 0.86, for social resources *α* = 0.83, and for social competence *α* = 0.75.

As a fifth measure, weight‐loss specific social support was assessed with a question included 1‐year post‐surgery: “Is there someone who does not support your efforts to change habits/follow the diet recommendations?”, with a yes or no response.

Five measures were used to assess “biopsychosocial surgical outcomes”. The first measure involved recording weight, which was obtained using a platform scale (Seca 635 III; 0–300 kg) at all timepoints. Net weight loss was calculated as percent total weight loss plus percentage of recurrent weight gain. Thus, the net weight loss indicates how much weight was effectively lost following the surgery. Percent total weight‐loss was calculated as follows: %TWL = [(Weight on the day of surgery) − (Postoperative Weight)]/(Weight on the day of surgery) × 100] [51]. The second measure, percent recurrent weight gain (as percent of pre‐surgery weight), was calculated as %WR = [(Postoperative Weight)‐(nadir weight)]/(Weight on the day of surgery) × 100]. The net weight loss and percent recurrent weight gain were then entered into the analyses.

The third measure was satisfaction with surgery. Satisfaction with surgery was measured 5 years post‐surgery with the question “To what extent were your expectations for the obesity surgery/treatment fulfilled?”, to be answered on a Likert‐scale with 5 options from “not at all” [1] to “wholly” [5]. The fourth measure was blood pressure, which was assessed with a CAS 730 Monitor, Ortomodic AS). Only systolic blood pressure was used for analysis, because it is typically highly correlated with diastolic blood pressure [52]. The difference in systolic blood pressure before surgery to 5 years post‐surgery was used for further analysis.

The fifth measure were symptoms of depression and anxiety, which were assessed with the Hospital Anxiety and Depression Scale (HADS) [53]. The subscales for anxiety and depression were reversed and summed up [54], capturing general psychological symptom burden (“distress”) [55]. Sum scores range from 0 to 42, and values of 19 or above indicate “probable” disorder. The HADS is a widely and internationally validated screening and evaluation instrument for mental health issues. The anxiety and depression subscales have shown adequate reliability with Cronbach's alpha above 0.70 in various applications of the Norwegian version [56]. The HADS also has sufficient sensitivity and specificity for detecting disorders. For the current study, the difference in (symptoms of) depression and anxiety before surgery to 5 years post‐surgery was entered into the analysis.

Expectations of surgery outcome served as potential covariate in the analysis because it was assumed that higher expectations might lead to lower satisfaction with the outcome of surgery. Patients' expectations regarding the impact of surgery on their lives were measured prior to surgery. Nine items were used that all started with “I will”, followed by different statements, to be answered on a scale from 1 to 10, with higher numbers indicating a larger agreement. The response on the statements “be happy with the amount of weight I have lost”, “be happy with my general appearance”, “have high self‐esteem”, “feel good”, were then averaged to form a score for “emotional expectations”. The response on the statements “have an increased work capacity (at home or in the work place)”, “have a better sex life”, “be a more successful person”, “be outgoing”, “have less to worry about”, were averaged to form a score for “expectations of optimal outcome”.

#### 2.2.2 Variables for Testing if Biopsychosocial Surgical Outcomes 1‐Year Post‐Surgery Predict Improved Social Relations 3 Years Post‐Surgery

Here the following three measures of biopsychosocial surgical outcomes were used as predictors: percent total weight loss (%TWL), depression and anxiety 1‐year post‐surgery, and satisfaction with weight loss. Satisfaction with weight loss was assessed 1‐year post‐surgery with the question “Are you satisfied with your weight loss so far?”, using a seven‐point Likert scale where higher values indicated greater satisfaction.

These predictors were used to address two variables measuring social relations. The first variable pertains to the difference in relationship satisfaction prior to surgery and 3 years post‐surgery, assessed using the RAS for participants who had a partner. This variable was not available for patients without a partner. Positive values indicate an increase in relationship satisfaction 3 years post‐surgery.

The second variable explores potential changes in relationships with friends for all participants. This was measured with the question “Think of your closest friends before surgery. How is your relationship with them now?» which was posed 3 years after surgery. Response options were: “I am no longer friends with one or more of them” (coded as 1), “We are still friends, but I'm not so close friends with one or more of them as I was before surgery” (coded as 2), “We are still friends, and I am equally close friend with whom I was before surgery” (coded as 3), “We are still friends, and I'm even closer with one or more of them now than I was before surgery” (coded as 4), so that higher values indicate an improvement of relationships. For analysis, this variable was dichotomized (codes 1‐2 = 0; codes 3‐4 = 1).

### 2.3 Analysis

All analyses were performed with the software R, version 4.3.1.

To assess potential attrition bias, a logistic regression model with participation as a binary outcome (1 for dropouts and 0 otherwise) and the pre‐surgery variables as predictors was calculated. The variable family coherence was dropped from this analysis because of multicollinearity issues.

Normality assumptions were checked for all outcome variables based on the Q‐Q plot of residuals and standardized residuals. The plots implied severe non‐normality. Hence, a non‐parametric bootstrapping approach was employed to test both hypotheses. Multicollinearity was checked for all covariates in all analyses by singular value decomposition of the model data matrix. There were no severe multicollinearity problems.

The randomness of missing values was tested via the Little's MCAR test (Chi‐Square = 683, DF = 652, Sig = 0.193). The null hypothesis that the missing data are completely at random was not rejected. Thus, using the R package mice, a model based multiple imputation approach was employed to handle missing data. The imputation of missing values was performed nested within each bootstrap resampled data as suggested by Schomaker and Heumann [57], and finally, all the results were pooled.

All the continuous variables were standardized (*z*‐scores).

#### 2.3.1 Analyses on the Role of Social Relations as a Predictor of Biopsychosocial Surgical Outcomes

Path analysis was employed to examine the relationships among different variables. Path analysis is based on regression models. Thus, a regression model for each outcome variable was fitted to the data using the R package lavaan. In path analysis, effects are categorized as direct (the main association of a predictor with the outcome), indirect (the association mediated through other variables), and total (the comprehensive effect combining direct and indirect associations). Path analysis also allows to detect relationships between different outcome variables.

Such a path model was applied to assess the relationships between the social relationship factors prior to surgery (relationship stability, relationship status, family coherence, social competence, social resources) and weight‐loss specific social support 1‐year post‐surgery as predictors, and the biopsychosocial outcomes 5 years post‐surgery (change in blood pressure, change in depression and anxiety, net weight loss, recurrent weight gain, and satisfaction with surgery) as outcome variables. Additionally, it was assumed that the biopsychosocial outcome variables are associated with each other, and that their relationships are potentially mediated by change in blood pressure and change in depression and anxiety 5 years post‐surgery. Accordingly, net weight loss and recurrent weight gain were also used as predictors for change in blood pressure, change in depression and anxiety, and satisfaction with surgery.

Gender, optimal outcome expectations prior to surgery, and emotional expectations pre‐surgery were included as covariates. Gender was used as a covariate due to established differences in mental health between men and women (e.g., [58]), and because research indicates that women exhibit distinct patterns in health behavior changes [59]. Optimal outcome expectations prior to surgery and emotional expectations prior to surgery were used as covariates because they might influence the relationship between social factors (relationship stability, relationship status, family coherence, social competence, social resources) and satisfaction with surgery.

Initially, a path model was fitted including relationship satisfaction for patients who had a partner prior to surgery only, because this variable was not available for patients without a partner. However, since relationship satisfaction was not significantly related to any of the outcome variables, a revised model without the variable relationship satisfaction was constructed. This allowed inclusion of patients without a partner. Multicollinearity was detected between the variables relationship status and family coherence, both measured prior to surgery. To address this issue, the main effect of relationship status was removed, and instead, the interaction effect between relationship status and family coherence was included as a covariate in the model.

The resulting model (see Supporting Information S1: Figure S1) was fitted to the data of all patients. This model was used to test the following secondary hypotheses: First, that relationship status, relationship stability, family coherence, social resources, social competence, and social support for weight loss are directly related to satisfaction. Patients in relationships, with better social resources, family coherence, social competence and weight‐loss specific social support, were hypothesized to be more satisfied with the outcome of the surgery. Second, that pre‐ surgery expectations are linked to satisfaction with surgery, with higher expectations being associated with lower satisfaction.

Additional analyses were performed to examine the relationships between various variables (see arrows in Supporting Information S1: Figure S1) that might potentially influence the primary relationship between social relationships and bariatric surgery outcomes, for example, the relationship between net weight loss and blood pressure. These results are reported for reasons of transparency, but they will not be discussed in the Discussion section, as they are not the focus of the study.

#### 2.3.2 Analyses on Biopsychosocial Surgical Outcomes as Predictors of Improved Social Relations

To test if biopsychosocial surgical outcomes predicted changes in social relations, a multiple regression was calculated for patients with partners, and a logistic regression was calculated for all patients.

##### 2.3.2.1 Analysis for Patients With Partners Only

For the multiple regression model, change of relationship satisfaction with the partner was the outcome. Weight loss (%TWL), satisfaction with weight loss and depression and anxiety 1‐ year post‐surgery were used as predictors. Side effects and gender were included as covariates in the analysis to adjust for their influence on partner relationship satisfaction. Negative side effects of the surgery were included as covariates because we previously found side effects to be important for satisfaction with bariatric surgery [60]. Negative side effects were measured one year after surgery with a questionnaire, where patients answered the question “Have you experienced any side effects/changes after the operation that have affected how you eat or being physically active?” for 11 different symptoms (e.g., dumping, pain) on a scale from 1 (no) to 6 (very much). The mean value from 7 negative side effects 1 year after surgery was calculated (fatigue, pain, dumping, diarrhea, constipation, heartburn, and vomiting).

##### 2.3.2.2 Analysis for all Patients

A logistic regression model was calculated for all patients, with changes in relationship with friends as outcome variable instead of partner relationship satisfaction.

## 3 Results

### 3.1 Participants

Attrition analysis did not show any systematic drop‐out effects (see Table 1).

The sample consisted of 58 men (20.14%) and 230 (79.86%) women. Their ages ranged from 20 to 73 years with a mean age of 43.90 and an interquartile range (IQR) between 37 and 51.

There were 202 patients (66.22%) who reported having a partner prior to surgery, while 103 patients (33.77%) were single. Five years post‐surgery, 160 patients (72.73%) had a partner and 60 patients (27.27%) were single. Out of 219 patients with complete data at both time points, 49 remained single from before surgery to five years after, with 6 having had a partner at some point during that period. A total of 138 patients maintained their partnered status throughout this time frame, although 7 changed partners and either cohabited or married the new partner. Additionally, 21 patients who were initially single found a partner by the 5‐year mark, while 11 who had a partner before the surgery became single during this period.

The descriptive statistics including mean, standard deviation (SD), minimum (Min.), maximum (Max.), the first quartile (Q1) and the third quartile (Q3) for the variables BMI and %TWL are reported in Tables 3 and 4, respectively.

**TABLE 3:** BMI at four time points (for patients with data at all four measurement time points).

Time point	Mean	SD	Min.	Max.	Q1	Q3
Prior to surgery	43.384	5.491	32.369	58.805	39.451	47.300
1 year post‐surgery	30.858	5.491	19.835	48.374	27.556	34.621
3 years post‐surgery	31.758	5.777	19.835	50.391	27.958	35.009
5 years post‐surgery	32.872	6.107	20.519	52.422	28.938	36.879

**TABLE 4:** %TWL at three time points (for patients with data at all three measurement time points).

Time point	Mean	SD	Min.	Max.	Q1	Q3
1 year post‐surgery	28.774	9.537	−38.462	53.600	23.795	34.141
3 years post‐surgery	26.695	9.933	5.470	54.143	19.909	33.840
5 years post‐surgery	24.097	10.783	3.409	52.000	17.179	30.414

Mean and standard deviation (SD) for several biopsychosocial variables measured pre‐surgery are reported in Table 5. Out of 282 patients, 209 patients exhibited depression and anxiety scores prior to surgery that met or exceeded the clinical cut‐off of 8.

**TABLE 5:** Pre‐surgery biopsychosocial variables.

Variable	Mean	SD
Depression and anxiety	12.362	7.058
Systolic blood pressure	141.021	17.373
Relationship satisfaction with partner	5.588	1.128
Family coherence	3.591	0.968
Social competence	3.686	0.826
Social resources	4.091	0.768
Emotional expectations	8.362	1.441
Optimal outcome expectations	7.536	1.655

The patients reported relatively high social resources and the standard deviations of family coherence, social competence and social resources indicate little variation in these variables.

### 3.2 Results on the Role of Social Relations Prior to Surgery as a Predictor of Biopsychosocial Surgical Outcomes 5 years Post‐Surgery

Of the variables defining “biopsychosocial surgical outcome”, satisfaction 5 years post‐surgery could be predicted from social resources prior to surgery (beta = 0.178, ci = [0.019,0.344]). Higher social support from people outside the family was associated with higher satisfaction. Furthermore, an increase in family coherence for patients with a partner was associated with a greater reduction in blood pressure 5 years post‐surgery (beta = −0.211, ci = [−0.424,−0.021]). Other social variables ‐ relationship stability, social competence, and social support of weight loss ‐ did not affect biopsychosocial surgical outcomes. For a detailed overview of the results, see Supporting Information S1: Table S1.

#### 3.2.1 Additional Results

Mean pre‐surgery blood pressure of 141 mmHg dropped to 132 mmHg after 5 years. Net weight loss was lower for men than women (beta = −0.319, ci = [−0.591,−0.043]). Net weight loss predicted lower depression and anxiety (beta = −0.139, ci = [−0.266,−0.007]), a greater reduction in blood pressure (beta = −0.207, ci = [−0.317,−0.088]), and higher satisfaction with surgery (total effect beta = 0.364, ci = [0.225,0.474]). The effect of net weight loss on satisfaction with surgery was partially mediated by depression and anxiety (direct effect beta = 0.296, ci = [0.148,0.435] and indirect effect beta = 0.032, ci = [0.017,0.051]). Since higher net weight loss is associated with lower depression and anxiety, this can partially explain the higher satisfaction. In other words, satisfaction with surgery is not only due to weight loss itself, but also to greater general well‐being. A decrease in depression and anxiety was also in itself, that is as a direct effect, related to higher satisfaction with surgery (beta = −0.136, ci = [−0.267,−0.021]). Moreover, greater recurrent weight gain was associated with higher depression and anxiety (beta = 0.124, ci = [0.014,0.241]).

### 3.3 Results on Biopsychosocial Surgical Outcomes 1‐Year Post‐Surgery as Predictors of Improved Social Relations 3 Years Post‐Surgery

Forty‐six patients were single both one year and three years post‐surgery; 120 patients had a partner 1 year and 3 years post‐surgery. Eleven patients were single 1‐year post‐surgery, but had a partner 5 years post‐surgery. Five patients had a partner 1 year post‐surgery, but had become single 3 years post‐surgery. Thus, structural changes in relationships had only occurred in 16 out of 162 patients.

There was no evidence that objective or subjective biopsychosocial surgical outcomes 1‐year post‐surgery improved social relations, either for relationships with partners (Table 6) or friends (Table 7). Depression and anxiety 1‐year post‐surgery were associated with decreased relationship satisfaction with the partner 3 years post‐surgery. Other factors such as side effects and gender did not affect changes in partner relationship satisfaction or changed relationships with friends.

**TABLE 6:** Multiple regression model on the relationship between biopsychosocial surgical outcomes and change in relationship satisfaction with partner. The brackets indicate that the category “man” was compared to the reference category ‘woman’. Significant effects are marked in bold font.

Predictor	Estimate	Std.Error	Ci.Lower	Ci.Upper
Intercept	−0.050	0.147	−0.322	0.244
Satisfaction with weight loss 1 year after surgery	−0.060	0.092	−0.254	0.110
**Depression and anxiety 1 year after surgery**	**−0.179**	**0.093**	**−0.370**	**−0.012**
Weight loss 1 year after surgery	0.002	0.088	−0.167	0.176
Side effects 1 year after surgery	0.066	0.089	−0.109	0.234
Gender [man]	−0.197	0.192	−0.595	0.172

**TABLE 7:** Logistic regression model on the relationship between biopsychosocial surgical outcomes and change in the relationship with friends. The brackets indicate that the category “man” was compared to the reference category ‘woman'.

Predictor	Estimate	Std.Error	Ci.Lower	Ci.Upper
Intercept	0.848	0.185	0.502	1.235
Satisfaction with weight loss 1 year after surgery	−0.032	0.187	−0.429	0.352
Depression and anxiety 1 year after surgery	−0.215	0.178	−0.590	0.161
Weight loss 1 year after surgery	−0.218	0.182	−0.599	0.136
Side effects 1 year after surgery	−0.076	0.189	−0.450	0.300
Gender [man]	0.390	0.381	−0.362	1.129

## 4 Discussion

The overarching aim of the study was to examine the relationship between social factors and indicators of physiological and mental well‐being in patients undergoing metabolic bariatric surgery. Specifically, the study aimed to determine whether social‐relationship factors predict broader biopsychosocial surgical outcomes, and if biopsychosocial surgical outcomes, in turn, predict enhanced social relationships.

### 4.1 Social Relations as a Predictor of Biopsychosocial Surgical Outcomes

Overall, limited evidence suggests a potential association between social factors and biopsychosocial surgical outcome. Social resources, defined as the availability and support of people outside the family, predicted higher satisfaction with surgery 5 years after surgery. It is possible that friends help patients adjust their expectations toward a more realistic view, as one of the main benefits of friendship is providing honest feedback ([61] p.197). Furthermore, an increase in family coherence was associated with a greater reduction in blood pressure 5 years post‐surgery, but only in patients with a partner. High family coherence has been associated with healthier food‐related behaviors in general [62]. Additionally, changes in health behaviors following bariatric surgery may influence family members to adopt healthier lifestyle practices. For instance, family members may actively support the patient's dietary modifications or engage in similar nutritional and physical activity changes, which can also lead to weight loss among family members [63]. A decrease in blood pressure may, therefore, be explained indirectly by the promotion of a healthier lifestyle within the family. Across many studies, family‐based social support has been linked to positive physiological outcomes, specifically demonstrating protective effects on cardiovascular functioning, hormonal regulation, and immune system responses. These beneficial effects appear to be independent of health‐related behaviors, indicating that family‐based social support may have direct physiological impacts beyond behavioral mechanisms [64]. Such physiological benefits may be mediated through social support's stress‐buffering effect [65].

Family coherence interacted with relationship status, as increased family coherence was linked to a greater reduction in blood pressure 5 years after surgery only among patients who had a partner. This could mean that the combined influence of having a partner and a cohesive family environment may have a stronger association with lower blood pressure levels compared to each factor on its own. A partner might provide additional emotional support and stress buffering that enhances the positive health effects of a cohesive family environment. Without a partner, individuals might not experience the same reduction in stress, potentially affecting blood pressure. By considering these two factors together, the impact on long‐term blood pressure outcomes may be more pronounced. These results underscore the importance of considering the interplay between social factors and health outcomes.

None of the factors relationship stability, social competence, or social support of weight loss ‐ had an effect on biopsychosocial surgical outcome. Additionally, relationship satisfaction with the partner did not seem to be important for biopsychosocial surgical outcome, as it was not related to any outcome variables in patients with a partner.

These findings partly differ from those reported in the literature. For patients who were married compared to singles, improved weight loss has been reported [26], but also no difference in weight loss [27], and lower weight loss [66]. In line with these discrepancies, a review concluded that the results regarding a potential link between structural relations and bariatric surgery outcomes were inconclusive [25]. Findings about social support appear to be more consistent [28, 29, 30], showing positive effects on surgery outcomes. The latter two studies indicate that relationship satisfaction plays an important role in the beneficial effects of marital and partner relationships. In contrast, our study did not find beneficial effects of relationship satisfaction. Altogether, social factors in our study only had a limited and selective contribution to biopsychosocial surgical outcome. One explanation is that social factors are of minor importance compared to the significant physical changes caused by the operation. However, this should also be true in other studies, where effects were still observed. Some of the differences between our study and other studies might be due to variation in time frames. In the studies using a follow‐up period of only of 2 years [26, 66] or 18 months [28], the stability of the relationships examined remains uncertain, as some of these relationships may have ended after the measurements were taken. Other reasons for diverging results could be variations in statistical methods, the use of diverse outcome variables, different covariates, and differing sample sizes.

Another possibility is that the patients in the current study generally had good or at least not particularly poor relationships. There was also little variation in reported family coherence, social resources, and social competence across patients, suggesting there might not have been enough variation in social support to show effects. However, further research with a comprehensive investigation of the variables is warranted to provide a more definitive conclusion.

### 4.2 Biopsychosocial Surgical Outcomes as Predictors of Improved Social Relations

Regarding the effect of surgery on social relationships, our data provided little support for this assumption. Structural changes in relationships were observed in only 10% of the patients. Moreover, changes in relationship satisfaction with a partner or changes in relationships with a friend were not related to weight loss. Only lower depression and anxiety 1‐year after surgery were associated with higher relationship satisfaction with the partner 3 years post‐surgery. Weight loss was not related to relationship satisfaction.

At first glance, our findings appear to contradict studies that demonstrate a relationship between bariatric surgery and both the structural and functional aspects of relationships. For instance, approximately 10 years post‐surgery, bariatric surgery patients exhibited a higher incidence of divorce, separation, marriage, and new partnerships compared to a control group [38]. It has also been reported that individuals with improved relationships, that is higher relationship quality post‐surgery, had a higher percentage of excess weight loss at roughly 8 years post‐surgery than those with constant or worsened relationships [30]. However, our study examined a shorter time frame than these studies, focusing on the relationship between weight loss 1 year after surgery and relationship satisfaction 3 years post‐surgery. Potentially, changes in relationships become more obvious across longer time periods.

Based on our findings, patients hoping to improve their existing relationships in the short‐term through weight loss achieved via surgery may be harboring unrealistic expectations.

### 4.3 Strengths and Limitations of the Current Study

One strength of our study is the 5‐year longitudinal design, which allows for tracking long‐term changes in patient outcomes and social factors. A further strength is the use of additional biopsychosocial outcome variables in addition to weight loss, all of which can contribute to quality of life [67, 68]. One limitation is incomplete data on relationship dynamics. Data on family coherence, social resources, social competence and relationship satisfaction were not collected at every time point. Furthermore, partner changes cannot be tracked definitely when participants report having a partner at consecutive time points. This highlights the need for more comprehensive data collection in future research on bariatric surgery outcomes.

### 4.4 Implications

The current findings are relevant for clinical practice. They indicate that, at least in patients with an “average” level of social support, social factors are not crucial for biopsychosocial surgical outcome. Therefore, healthcare providers might focus on other factors that have shown a greater impact. This knowledge can help guide the development and implementation of interventions targeting those areas such as preoperative preparation, postoperative care, and psychological support. However, more research is required to draw any definitive conclusions about the relevance or irrelevance of social factors for biopsychosocial surgical outcome.

Additionally, patients and their families often have high expectations for the impact of metabolic bariatric surgery on social relationships. Sharing the findings of this study can help set more realistic expectations and prevent unwarranted disappointment or frustration if improvements in social relationships are not observed after surgery. Additional support and intervention may be necessary for those who wish to improve social integration and quality of relationships post‐surgery.

## Conflicts of Interest

The authors declare no conflicts of interest.

## Supporting information

Supporting Information S1

## References

[1] J. S. House , K. R. Landis , and D. Umberson , “Social Relationships and Health,” Science 241, no. 4865 (1988): 540–545, 10.1126/science.3399889. 3399889

[2] F. Wang , Y. Gao , Z. Han , et al., “A Systematic Review and Meta‐Analysis of 90 Cohort Studies of Social Isolation, Loneliness and Mortality,” Nature Human Behaviour 7, no. 8 (2023): 1307–1319, 10.1038/s41562-023-01617-6. 37337095

[3] L. C. Hawkley , M. E. Hughes , L. J. Waite , C. M. Masi , R. A. Thisted , and J. T. Cacioppo , “From Social Structural Factors to Perceptions of Relationship Quality and Loneliness: The Chicago Health, Aging, and Social Relations Study,” Journal of Gerontology: Series B 63, no. 6 (2008): S375–S384, 10.1093/geronb/63.6.s375. PMC2769562 19092047

[4] A. F. Danvers , L. D. Efinger , M. R. Mehl , et al., “Loneliness and Time Alone in Everyday Life: A Descriptive‐Exploratory Study of Subjective and Objective Social Isolation,” Journal of Research in Personality 107 (2023): 104426, 10.1016/j.jrp.2023.104426.

[5] D. Perlman and L. A. Peplum , “Toward a Social Psychology of Loneliness,” in Personal Relationships: 3 Relationships in Disorder (Academic Press, 1981), 31–56.

[6] H. Reis and S. Sprecher , “Loneliness,” in Encyclopedia of Human Relationships [Internet] (SAGE Publications, Inc., 2009), 986–990, https://sk.sagepub.com/reference/humanrelationships/n318.xml.

[7] C. Fekete , H. Tough , M. Arora , et al., “Are Social Relationships an Underestimated Resource for Mental Health in Persons Experiencing Physical Disability? Observational Evidence From 22 Countries,” International Journal of Public Health 66 (2021): 619823, 10.3389/ijph.2021.619823. 34744581 PMC8565297

[8] C. Zürcher , H. Tough , C. Fekete , and S. S. Group for the , “Mental Health in Individuals With Spinal Cord Injury: The Role of Socioeconomic Conditions and Social Relationships,” PLoS One 14, no. 2 (2019): e0206069, 10.1371/journal.pone.0206069. 30785880 PMC6382129

[9] V. S. Helgeson , “Social Support and Quality of Life,” Quality of Life Research 12, no. 1 (2003): 25–31, 10.1023/a:1023509117524. 12803308

[10] L. C. Hawkley , R. A. Thisted , and J. T. Cacioppo , “Loneliness Predicts Reduced Physical Activity: Cross‐Sectional & Longitudinal Analyses,” Health Psychology 28, no. 3 (2009): 354–363. 19450042 10.1037/a0014400 PMC2791498

[11] L. C. Kobayashi and A. Steptoe , “Social Isolation, Loneliness, and Health Behaviors at Older Ages: Longitudinal Cohort Study,” Annals of Behavioral Medicine 52, no. 7 (2018): 582–593, 10.1093/abm/kax033. 29860361 PMC6377432

[12] K. Hanna , J. Cross , A. Nicholls , and D. Gallegos , “The Association Between Loneliness or Social Isolation and Food and Eating Behaviours: A Scoping Review,” Appetite 191 (2023): 107051, 10.1016/j.appet.2023.107051. 37802217

[13] X. Zhang , S. Ravichandran , G. C. Gee , et al., “Social Isolation, Brain Food Cue Processing, Eating Behaviors, and Mental Health Symptoms,” JAMA Network Open 7, no. 4 (2024): e244855, 10.1001/jamanetworkopen.2024.4855. 38573637 PMC11192185

[14] Y. Yu , Q. Huang , Z. Ren , and Z. Ou , “The Association Between Social Isolation and Medication Adherence Among Chinese Older Adults With Chronic Diseases: Serial Mediation of Social Support and Loneliness,” Annals of Behavioral Medicine 58, no. 10 (2024 Oct 16): 670–678, 10.1093/abm/kaae049. 39158009

[15] W. Lauder , K. Mummery , M. Jones , and C. Caperchione , “A Comparison of Health Behaviours in Lonely and Non‐lonely Populations,” Psychology Health & Medicine 11, no. 2 (2006): 233–245, 10.1080/13548500500266607. 17129911

[16] J. F. Schumaker , R. C. Krejci , L. Small , and R. G. Sargent , “Experience of Loneliness by Obese Individuals,” supplement, Psychological Reports 57, no. S3 (1985): S1147–S1154, 10.2466/pr0.1985.57.3f.1147. 4095228

[17] J. Zhou , R. Tang , X. Wang , X. Li , Y. Heianza , and L. Qi , “Improvement of Social Isolation and Loneliness and Excess Mortality Risk in People With Obesity,” JAMA Network Open 7, no. 1 (2024): e2352824, 10.1001/jamanetworkopen.2023.52824. 38252435 PMC10804268

[18] H. Buchwald , Y. Avidor , E. Braunwald , et al., “Bariatric Surgery: A Systematic Review and Meta‐Analysis,” JAMA 292, no. 14 (2004): 1724–1737, 10.1001/jama.292.14.1724. 15479938

[19] T. H. Inge , L. M. Laffel , T. M. Jenkins , et al., “Comparison of Surgical and Medical Therapy for Type 2 Diabetes in Severely Obese Adolescents,” JAMA Pediatrics 172, no. 5 (2018): 452–460, 10.1001/jamapediatrics.2017.5763. 29532078 PMC5875354

[20] P. Salminen , S. Grönroos , M. Helmiö , et al., “Effect of Laparoscopic Sleeve Gastrectomy vs Roux‐En‐Y Gastric Bypass on Weight Loss, Comorbidities, and Reflux at 10 Years in Adult Patients With Obesity: The SLEEVEPASS Randomized Clinical Trial,” JAMA Surg 157, no. 8 (2022): 656–666, 10.1001/jamasurg.2022.2229. 35731535 PMC9218929

[21] L. Sjöström , A. K. Lindroos , M. Peltonen , et al., “Lifestyle, Diabetes, and Cardiovascular Risk Factors 10 Years After Bariatric Surgery,” New England Journal of Medicine 351, no. 26 (2004): 2683–2693, 10.1056/nejmoa035622. 15616203

[22] K. J. Coleman , Y. C. Huang , F. Hendee , H. L. Watson , R. A. Casillas , and J. Brookey , “Three‐Year Weight Outcomes From a Bariatric Surgery Registry in a Large Integrated Healthcare System,” Surg Obes Relat Dis Off J Am Soc Bariatr Surg 10, no. 3 (2014): 396–403, 10.1016/j.soard.2014.02.044. 24951065

[23] J. Himpens , J. Dobbeleir , and G. Peeters , “Long‐term Results of Laparoscopic Sleeve Gastrectomy for Obesity,” Annals of Surgery 252, no. 2 (2010): 319–324, 10.1097/sla.0b013e3181e90b31. 20622654

[24] S. Karmali , B. Brar , X. Shi , A. M. Sharma , C. de Gara , and D. W. Birch , “Weight Recidivism Post‐bariatric Surgery: A Systematic Review,” Obesity Surgery 23, no. 11 (2013): 1922–1933, 10.1007/s11695-013-1070-4. 23996349

[25] M. Livhits , C. Mercado , I. Yermilov , et al., “Is Social Support Associated With Greater Weight Loss After Bariatric Surgery?: A Systematic Review,” Obes Rev Off J Int Assoc Study Obes. 12, no. 2 (2011): 142–148, 10.1111/j.1467-789x.2010.00720.x. 20158617

[26] S. Wedin , A. Madan , J. Correll , et al., “Emotional Eating, Marital Status and History of Physical Abuse Predict 2‐year Weight Loss in Weight Loss Surgery Patients,” Eating Behaviors 15, no. 4 (2014): 619–624, 10.1016/j.eatbeh.2014.08.019. 25241076

[27] M. Ferber , L. M. Hecht , K. M. Martens , A. Hamann , A. M. Carlin , and L. R. Miller‐Matero , “Examining Differences in Long‐Term Weight Loss Outcomes After Bariatric Surgery: The Role of Romantic Relationship Status,” Families, Systems & Health 42, no. 1 (2024): 122–126, 10.1037/fsh0000832. 37616105

[28] E. M. Conceição , M. Fernandes , M. de Lourdes , A. Pinto‐Bastos , A. R. Vaz , and S. Ramalho , “Perceived Social Support Before and After Bariatric Surgery: Association With Depression, Problematic Eating Behaviors, and Weight Outcomes,” Eat Weight Disord EWD 25, no. 3 (2020): 679–692, 10.1007/s40519-019-00671-2. 30859467

[29] E. Y. Q. Tan , P. C. Lee , K. W. Tham , S. Ganguly , C. H. Lim , and J. C. J. Liu , “Examining Spousal and Family Support as Predictors of Long‐Term Weight Loss and Remission of Type 2 Diabetes Following Bariatric Surgery in Singapore: A Retrospective Cohort Study,” BMJ Open 13, no. 5 (2023): e068810, 10.1136/bmjopen-2022-068810. PMC10163544 37130681

[30] S. M. Clark , K. K. Saules , L. M. Schuh , J. Stote , and D. B. Creel , “Associations Between Relationship Stability, Relationship Quality, and Weight Loss Outcomes Among Bariatric Surgery Patients,” Eating Behaviors 15, no. 4 (2014): 670–672, 10.1016/j.eatbeh.2014.09.003. 25308799

[31] D. I. Athanasiadis , R. A. Carr , C. Smith , et al., “Social Support provided to Bariatric Surgery Patients through a Facebook Group May Improve Weight Loss Outcomes,” Surgical Endoscopy 36, no. 10 (2022): 7652–7655, 10.1007/s00464-022-09067-3. 35182215 PMC8857391

[32] U. Tymoszuk , M. Kumari , A. Pucci , et al., “Is Pre‐Operation Social Connectedness Associated With Weight Loss up to 2 Years Post Bariatric Surgery?,” Obesity Surgery 28, no. 11 (2018): 3524–3530, 10.1007/s11695-018-3378-6. 30043144

[33] J. Ogden , A. Hollywood , and C. Pring , “The Impact of Psychological Support on Weight Loss Post Weight Loss Surgery: A Randomised Control Trial,” Obesity Surgery 25, no. 3 (2015): 500–505, 10.1007/s11695-014-1428-2. 25200170 PMC4346662

[34] E. Ahlich , J. B. Herr , K. Thomas , D. T. Segarra , and D. Rancourt , “A Test of the Stress‐Buffering Hypothesis of Social Support Among Bariatric Surgery Patients,” Surgery for Obesity and Related Diseases 16, no. 1 (2020): 90–98, 10.1016/j.soard.2019.10.020. 31813776

[35] S. E. Stromberg , R. Gonzalez‐Louis , M. Engel , A. Mathews , and D. M. Janicke , “Pre‐surgical Stress and Social Support Predict Post‐surgical Percent Excess Weight Loss in a Population of Bariatric Surgery Patients,” Psychology Health & Medicine 25, no. 10 (2020): 1258–1265, 10.1080/13548506.2020.1734216. 32101050

[36] A. Hawke , P. O’Brien , J. M. Watts , et al., “Psychosocial and Physical Activity Changes After Gastric Restrictive Procedures for Morbid Obesity,” Australian and New Zealand Journal of Surgery 60, no. 10 (1990): 755–758, 10.1111/j.1445-2197.1990.tb07469.x. 2206119

[37] C. S. W. Rand , J. M. Kuldau , and L. Robbins , “Surgery for Obesity and Marriage Quality,” JAMA 247, no. 10 (1982): 1419–1422, 10.1001/jama.1982.03320350023021. 7057530

[38] G. Bruze , T. E. Holmin , M. Peltonen , et al., “Associations of Bariatric Surgery With Changes in Interpersonal Relationship Status,” JAMA Surg 153, no. 7 (2018): 654–661, 10.1001/jamasurg.2018.0215. 29590289 PMC5875335

[39] K. L. Applegate and K. E. Friedman , “The Impact of Weight Loss Surgery on Romantic Relationships,” Bariatric Nursing and Surgical Patient Care 3, no. 2 (2008): 135–141, 10.1089/bar.2008.9976.

[40] J. R. Neill , J. R. Marshall , and C. E. Yale , “Marital Changes After Intestinal Bypass Surgery,” JAMA 240, no. 5 (1978): 447–458, 10.1001/jama.1978.03290050037013. 660889

[41] H. Konttinen , K. Sjöholm , L. M. S. Carlsson , M. Peltonen , and P. A. Svensson , “Fifteen‐Year Changes in Health‐Related Quality of Life After Bariatric Surgery and Non‐Surgical Obesity Treatment,” International Journal of Obesity 48, no. 10 (2024): 1447–1456. 38902388 10.1038/s41366-024-01572-w PMC11420074

[42] H. Ø Lier , S. Aastrom , and K. Rørtveit , “Patients’ Daily Life Experiences Five Years After Gastric Bypass Surgery‐‐A Qualitative Study,” Journal of Clinical Nursing 25, no. 3–4 (2016): 322–331. 26621613 10.1111/jocn.13049

[43] S. Azizi , N. Memaryan , K. Alavi , R. Sedigh , and A. Jolfaei Ghanbari , “A Qualitative Study on Patients’ Experiences of Interpersonal Relationships After Bariatric Surgery,” ijpcp 26, no. 1 (2020): 102–113.

[44] I. Ficaro , “Surgical Weight Loss as a Life‐Changing Transition: The Impact of Interpersonal Relationships on Post Bariatric Women,” Appl Nurs Res ANR 40 (2018): 7–12, 10.1016/j.apnr.2017.12.003. 29579502

[45] I. Bergh , I. L. Kvalem , H. Risstad , L. D. Cameron , and F. F. Sniehotta , “Predictors of Preoperative Weight Loss in Morbidly Obese Adults Waiting for Bariatric Surgery: A Prospective Cohort Study,” Obesity Surgery 25, no. 9 (2015): 1610–1617, 10.1007/s11695-015-1569-y. 25616397

[46] I. L. Kvalem , I. Bergh , T. von Soest , et al., “A Comparison of Behavioral and Psychological Characteristics of Patients Opting for Surgical and Conservative Treatment for Morbid Obesity,” BMC Obes 3, no. 1 (2016): 6, 10.1186/s40608-016-0084-6. 26885374 PMC4743365

[47] S. S. Hendrick , “A Generic Measure of Relationship Satisfaction,” Journal of Marriage and Family 50, no. 1 (1988): 93–98, 10.2307/352430.

[48] G. B. Spanier , “Measuring Dyadic Adjustment: New Scales for Assessing the Quality of Marriage and Similar Dyads,” Journal of Marriage and Family 38, no. 1 (1976): 15–28, 10.2307/350547.

[49] O. Friborg , M. Martinussen , and J. H. Rosenvinge , “Likert‐based vs. Semantic Differential‐Based Scorings of Positive Psychological Constructs: A Psychometric Comparison of Two Versions of a Scale Measuring Resilience,” Personality and Individual Differences 40, no. 5 (2006): 873–884, 10.1016/j.paid.2005.08.015.

[50] O. Hjemdal , A. Roazzi , M. da G. B. B. Dias , and O. Friborg , “The Cross‐Cultural Validity of the Resilience Scale for Adults: A Comparison Between Norway and Brazil,” BMC Psychology 3, no. 1 (2015): 18, 10.1186/s40359-015-0076-1. 26090106 PMC4471935

[51] S. A. Brethauer , J. Kim , M. el Chaar , et al., “Standardized Outcomes Reporting in Metabolic and Bariatric Surgery,” Surgery of Obestity and Related Diseases 11, no. 3 (2015): 489–506, 10.1016/j.soard.2015.02.003. 26093765

[52] B. Gavish , I. Z. Ben‐Dov , and M. Bursztyn , “Linear Relationship Between Systolic and Diastolic Blood Pressure Monitored Over 24 H: Assessment and Correlates,” Journal of Hypertension 26, no. 2 (2008): 199–209, 10.1097/hjh.0b013e3282f25b5a. 18192832

[53] A. S. Zigmond and R. P. Snaith , “The Hospital Anxiety and Depression Scale,” Acta Psychiatrica Scandinavica 67, no. 6 (June 1983): 361–370, 10.1111/j.1600-0447.1983.tb09716.x. 6880820

[54] S. C. Palmer , “Hospital Anxiety Depression Scale,” in Encyclopedia of Behavioral Medicine [Internet], eds. M. D. Gellman and J. R. Turner , (Springer New York, 2013), 988–989, 10.1007/978-1-4419-1005-9_962.

[55] T. D. Cosco , F. Doyle , M. Ward , and H. McGee , “Latent Structure of the Hospital Anxiety and Depression Scale: A 10‐year Systematic Review,” Journal of Psychosomatic Research 72, no. 3 (2012): 180–184, 10.1016/j.jpsychores.2011.06.008. 22325696

[56] K. A. Leiknes , T. K. Dalsbø , and J. Siqveland , Psychometric Assessment of the Norwegian Version of the Hospital Anxiety and Depression Scale (HADS). (Folkehelseinstituttet, 2016): (Rapport 2016).

[57] M. Schomaker and C. Heumann , “Bootstrap Inference When Using Multiple Imputation,” Statistics in Medicine 37, no. 14 (2018): 2252–2266, 10.1002/sim.7654. 29682776 PMC5986623

[58] M. Pilar Matud , “Gender and Health,” in Gender Differences in Different Contexts [Internet], ed. A. Alvinius , (IntechOpen, 2017): Ch. 4, 10.5772/65410.

[59] A. R. Assaf , D. Parker , K. L. Lapane , E. Coccio , E. Evangelou , and R. A. Carleton , “Does the Y Chromosome Make a Difference? Gender Differences in Attempts to Change Cardiovascular Disease Risk Factors,” J Womens Health 12, no. 4 (2003): 321–330, 10.1089/154099903765448835. 12804339

[60] I. Lundin Kvalem , L. Gabrielsen , I. Eribe , J. A. Kristinsson , and T. Mala , “Predicting Satisfaction With Outcome and Follow‐Up Care 5 Years After Bariatric Surgery: A Prospective Evaluation,” Obes Sci Pract 8, no. 5 (2022): 595–602, 10.1002/osp4.594. 36238221 PMC9535663

[61] Cicero. On Old Age , On Friendship. On Divination. Translated by W. A. Falconer (Harvard University Press, 1923): (Loeb Classical Library 154).

[62] J. Martin‐Biggers , V. Quick , M. Zhang , Y. Jin , and C. Byrd‐Bredbenner , “Relationships of Family Conflict, Cohesion, and Chaos in the Home Environment on Maternal and Child Food‐Related Behaviours,” Maternal and Child Nutrition 14, no. 2 (2018): e12540, 10.1111/mcn.12540. 28994511 PMC6866091

[63] G. A. Woodard , B. Encarnacion , J. Peraza , T. Hernandez‐Boussard , and J. Morton , “Halo Effect for Bariatric Surgery: Collateral Weight Loss in Patients’ Family Members,” Archives of Surgery 146, no. 10 (2011): 1185–1190, 10.1001/archsurg.2011.244. 22006878

[64] B. N. Uchino , “Social Support and Health: A Review of Physiological Processes Potentially Underlying Links to Disease Outcomes,” Journal of Behavioral Medicine 29, no. 4 (2006): 377–387, 10.1007/s10865-006-9056-5. 16758315

[65] K. S. Bowen , B. N. Uchino , W. Birmingham , M. Carlisle , T. W. Smith , and K. C. Light , “The Stress‐Buffering Effects of Functional Social Support on Ambulatory Blood Pressure,” Health Psychol Off J Div Health Psychol Am Psychol Assoc. 33, no. 11 (2014): 1440–1443, 10.1037/hea0000005. PMC4090296 24245843

[66] R. Lutfi , A. Torquati , N. Sekhar , and W. O. Richards , “Predictors of Success After Laparoscopic Gastric Bypass: A Multivariate Analysis of Socioeconomic Factors,” Surgical Endoscopy 20, no. 6 (2006): 864–867, 10.1007/s00464-005-0115-8. 16738971

[67] E. H. Ha , S. H. Lee , J. Jeong , et al., “Biopsychosocial Predictors of the Quality of Life in Breast Cancer Patients,” Journal of Breast Cancer 13, no. 2 (2010): 219–226, 10.4048/jbc.2010.13.2.219.

[68] M. Kojima , T. Kojima , N. Ishiguro , et al., “Psychosocial Factors, Disease Status, and Quality of Life in Patients With Rheumatoid Arthritis,” Journal of Psychosomatic Research 67, no. 5 (2009): 425–431, 10.1016/j.jpsychores.2009.01.001. 19837205

