# Supplementary material for: The Interplay Between Social Factors and Metabolic Bariatric Surgery Outcomes—A 5‐Year Prospective Study
Source: Obes Sci Pract. 2025 Mar 24;11(2):e70068. doi: 10.1002/osp4.70068 (PMC11931256; doi:10.1002/osp4.70068)
Supplement: Supplementary file 1 — Supporting Information S1 [file OSP4-11-e70068-s001.docx]

**Supplementary Materials of the article “The interplay between social factors and metabolic bariatric surgery outcomes - a 5-year prospective study”**


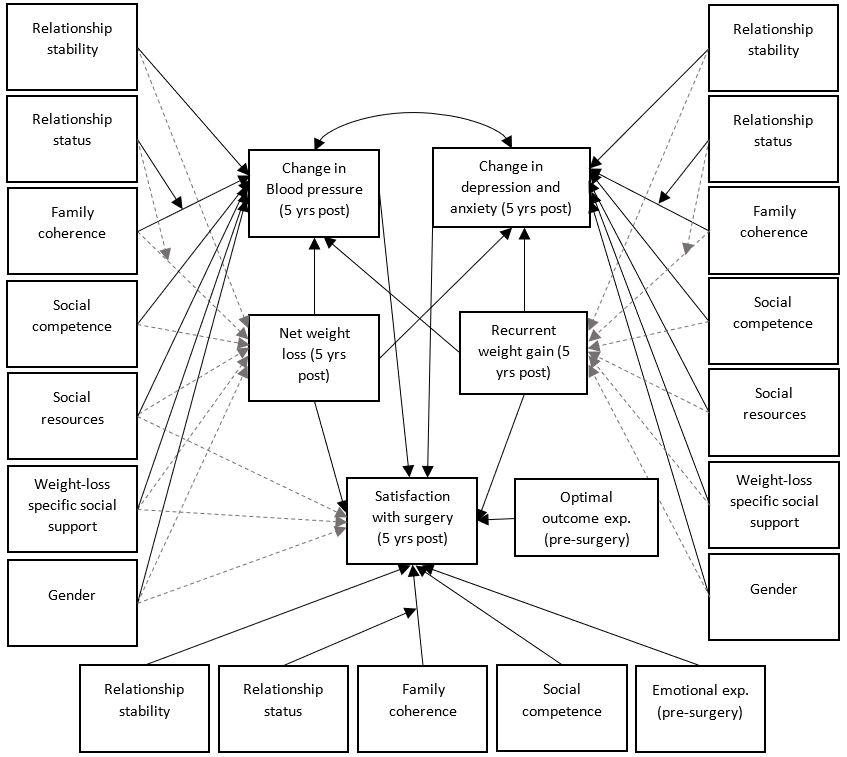


*Figure S1: Path model on the relationship between social factors and later biopsychosocial surgical outcomes. “Exp.” stands for “expectation”. The two-headed arrow indicates covariance between variables (how they vary together), and single-headed arrows indicate regression relationships (how one variable predicts another). Note that black and grey dashed arrows convey the same regression relationships; the use of grey dashed arrows is solely to reduce visual clutter in the graph.*

*Table S1: Relationship between social relations prior to surgery and biopsychosocial surgical outcomes* *5 years post-surgery (Ci = confidence interval). Significant effects are marked in bold font. Gender, relationship status, and weight-loss specific social support are represented as categorical variables. The brackets indicate the specific category being compared to a baseline or reference category. For the categorical variables, the reference categories are: 'woman' for gender, 'changing' for relationship stability, and 'no support' for weight loss-specific social support.*

| Outcome | Mediator | Predictor | Estimate | Std. Error | Ci. Lower | Ci. Upper |
| --- | --- | --- | --- | --- | --- | --- |
| Change in depression and anxiety 5 years after surgery | - | **Net weight loss 5 years after surgery** | **-0.139** | **0.068** | **-0.266** | **-0.007** |
|  |  | **Recurrent weight gain 5 years after surgery** | **0.124** | **0.058** | **0.014** | **0.241** |
|  |  | Family coherence for patients with partner | -0.120 | 0.142 | -0.388 | 0.159 |
|  |  | Family coherence for single patients | 0.143 | 0.127 | -0.097 | 0.405 |
|  |  | Relationship stability [with partner at baseline and 5 years after surgery] | -0.010 | 0.247 | -0.460 | 0.459 |
|  |  | Relationship stability [single at baseline and 5 years after surgery] | 0.047 | 0.152 | -0.247 | 0.336 |
|  |  | Social competence | 0.133 | 0.072 | -0.006 | 0.272 |
|  |  | Social resources | 0.075 | 0.077 | -0.076 | 0.227 |
|  |  | Weight-loss specific social support [with support] | 0.161 | 0.251 | -0.335 | 0.621 |
|  |  | Gender [man] | 0.112 | 0.146 | -0.161 | 0.393 |
| Change in systolic blood pressure 5 years after surgery | - | **Net weight loss 5 years after surgery** | **-0.207** | **0.058** | **-0.317** | **-0.088** |
|  |  | Recurrent weight gain 5 years after surgery | 0.043 | 0.053 | -0.061 | 0.149 |
|  |  | **Family coherence for patients with partner** | **-0.211** | **0.110** | **-0.424** | **-0.021** |
|  |  | Family coherence for single patients | 0.025 | 0.097 | -0.166 | 0.207 |
|  |  | Relationship stability [with partner at baseline and 5 years after surgery] | 0.253 | 0.186 | -0.072 | 0.634 |
|  |  | Relationship stability [single at baseline and 5 years after surgery] | -0.019 | 0.184 | -0.362 | 0.322 |
|  |  | Social competence | -0.014 | 0.070 | -0.146 | 0.121 |
|  |  | Social resources | 0.029 | 0.080 | -0.137 | 0.180 |
|  |  | Weight-loss-specific social support [with support] | 0.233 | 0.209 | -0.156 | 0.646 |
|  |  | Gender [man] | -0.094 | 0.138 | -0.373 | 0.173 |
| Net weight loss 5 years after surgery | - | Family coherence for patients with partner | -0.071 | 0.118 | -0.293 | 0.148 |
|  |  | Family coherence for single patients | -0.034 | 0.113 | -0.249 | 0.179 |
|  |  | Relationship stability [with partner at baseline and 5 years after surgery] | 0.304 | 0.214 | -0.127 | 0.700 |
|  |  | Relationship stability [single at baseline and 5 years after surgery] | 0.000 | 0.177 | -0.366 | 0.343 |
|  |  | Social competence | -0.026 | 0.067 | -0.160 | 0.101 |
|  |  | Social resources | -0.012 | 0.065 | -0.142 | 0.114 |
|  |  | Weight-loss-specific social support [with support] | -0.163 | 0.204 | -0.562 | 0.254 |
|  |  | **Gender [man]** | **-0.319** | **0.143** | **-0.591** | **-0.043** |
| Recurrent weight gain 5 years after surgery | - | Family coherence for patients with partner | 0.134 | 0.090 | -0.050 | 0.314 |
|  |  | Family coherence for single patients | -0.153 | 0.087 | -0.312 | 0.009 |
|  |  | Relationship stability [with partner at baseline and 5 years after surgery] | -0.047 | 0.150 | -0.322 | 0.255 |
|  |  | Relationship stability [single at baseline and 5 years after surgery] | 0.141 | 0.187 | -0.217 | 0.498 |
|  |  | Social competence | -0.062 | 0.059 | -0.180 | 0.046 |
|  |  | Social resources | -0.006 | 0.063 | -0.128 | 0.107 |
|  |  | Weight-loss-specific social support [with support] | 0.316 | 0.215 | -0.110 | 0.724 |
|  |  | Gender [man] | -0.128 | 0.154 | -0.426 | 0.186 |
| Satisfaction with surgery 5 years after surgery | - | **Net weight loss 5 years after surgery** | **0.296** | **0.075** | **0.148** | **0.435** |
|  |  | Recurrent weight gain 5 years after surgery | -0.090 | 0.051 | -0.197 | 0.004 |
|  |  | Change in systolic blood pressure 5 years after surgery | -0.182 | 0.176 | -0.498 | 0.214 |
|  | Change in depression and anxiety 5 years after surgery | **Net weight loss 5 years after surgery (indirect effect)** | **0.032** | **0.014** | **0.017** | **0.051** |
|  |  | **Net weight loss 5 years after surgery (total effect)** | **0.328** | **0.072** | **0.176** | **0.460** |
|  |  | Recurrent weight gain 5 years after surgery (indirect effect) | -0.016 | 0.012 | -0.043 | 0.002 |
|  |  | Recurrent weight gain 5 years after surgery (total effect) | -0.107 | 0.051 | -0.220 | -0.011 |
|  | Change in systolic blood pressure 5 years after surgery | Net weight loss 5 years after surgery (indirect effect) | 0.036 | 0.038 | -0.047 | 0.109 |
|  |  | **Net weight loss 5 years after surgery (total effect)** | **0.332** | **0.066** | **0.197** | **0.465** |
|  |  | Recurrent weight gain 5 years after surgery (indirect effect) | -0.008 | 0.015 | -0.043 | 0.017 |
|  |  | Recurrent weight gain 5 years after surgery (total effect) | -0.098 | 0.052 | -0.204 | 0.006 |
|  | - | **Change in depression and anxiety 5 years after surgery** | **-0.136** | **0.069** | **-0.267** | **-0.021** |
|  |  | Family coherence for patients with partner | 0.007 | 0.126 | -0.242 | 0.262 |
|  |  | Family coherence for single patients | -0.009 | 0.115 | -0.223 | 0.219 |
|  |  | Relationship stability [with partner at baseline and 5 years after surgery] | 0.103 | 0.210 | -0.316 | 0.525 |
|  |  | Relationship stability [single at baseline and 5 years after surgery] | 0.226 | 0.167 | -0.095 | 0.557 |
|  |  | Social competence | -0.087 | 0.068 | -0.212 | 0.064 |
|  |  | **Social resources** | **0.178** | **0.079** | **0.019** | **0.344** |
|  |  | Emotional expectations prior to surgery | 0.059 | 0.082 | -0.106 | 0.211 |
|  |  | Expectations of optimal outcome prior to surgery | -0.046 | 0.080 | -0.200 | 0.109 |
|  |  | Weight-loss-specific social support [with support] | -0.041 | 0.181 | -0.421 | 0.321 |
|  |  | Gender [man] | 0.242 | 0.157 | -0.070 | 0.540 |
